# Galerkin finite element study for mixed convection (TiO_2_–SiO_2_/water) hybrid-nanofluidic flow in a triangular aperture heated beneath

**DOI:** 10.1038/s41598-021-02216-z

**Published:** 2021-11-25

**Authors:** Fares Redouane, Wasim Jamshed, S. Suriya Uma Devi, M. Prakash, Kottakkaran Sooppy Nisar, Nor Ain Azeany Mohd Nasir, M. Motawi Khashan, I. S. Yahia, Mohamed R. Eid

**Affiliations:** 1 LGIDD, Ahmed ZABANA University, 48000 Relizane, Algeria; 2 Department of Mathematics, Capital University of Science and Technology (CUST), Islamabad, 44000 Pakistan; 3 Department of Mathematics, KPR Institute of Engineering and Technology, Coimbatore, 641407 India; 4 Department of Mathematics, Dr. N.G.P Institute of Technology, Coimbatore, 641407 India; 5 Department of Mathematics, College of Arts and Sciences, Prince Sattam Bin Abdulaziz University, Wadi Aldawaser, 11991 Saudi Arabia; 6 Department of Mathematics, Centre for Defence Foundation Studies, Universiti Pertahanan Nasional Malaysia, Kem Sungai Besi, 57000 Kuala Lumpur, Malaysia; 7 Department of Basic Sciences, Common First Year, King Saud University, Riyadh, 11451 Saudi Arabia; 8 Advanced Functional Materials and Optoelectronic Laboratory (AFMOL), Department of Physics, Faculty of Science, King Khalid University, P.O. Box 9004, Abha, Saudi Arabia; 9 Research Center for Advanced Materials Science (RCAMS), King Khalid University, P.O. Box 9004, Abha, 61413 Saudi Arabia; 10 Nanoscience Laboratory for Environmental and Biomedical Applications (NLEBA), Semiconductor Lab., Department of Physics, Faculty of Education, Ain Shams University, Roxy, Cairo 11757 Egypt; 11 Department of Mathematics, Faculty of Science, New Valley University, Al-Kharga, Al-Wadi Al-Gadid 72511 Egypt; 12 Department of MathematicsFaculty of Science, Northern Border University, Arar, 1321 Saudi Arabia

**Keywords:** Mathematics and computing, Physics

## Abstract

Fluidity and thermal transport across the triangular aperture with lower lateral inlet and apply placed at the vertical outlet of the chamber which filled with efficient TiO_2_–SiO_2_/water hybrid nanofluid under the parametrical influence. Several parameters are tested like the numbers of Hartmann ($0 \le Ha \le 100$), Richardson ($0 \le Ri \le 5$), and Reynolds ($10 \le Re \le 1000$) were critiqued through streamlines, isotherms, and Nusselt number ($Nu$). Numerical model has to be developed and solved through the Galerkin finite element method (GFEM) by discretized with 13,569 triangular elements optimized through grid-independent analysis. The Hartmann number ($Ha$), exerts minimal impact over the flow and thermal aspects while the other parameters significantly manipulate the physical nature of the flowing and thermal aspects behaviors.

## Introduction

Nanofluids, an alluring word that fascinates thermal-based researchers over the decades. Its astounding combination of efficiency and applicability makes it the choice at the first palace. Starts from basic electrical and electronic utilities its eminence branched out in biomedical, automobile and even reached the space technology^1–6^. In 1995, Choi^7^ was the foremost scientist to float on a new nanofluid theory. A nanofluid is known as the colloidal suspension in the traditional fluid of metal (H_2_O, C_2_H_6_O_2_, and other base fluids) nanosized (< 100 nm) which places the nanofluid as the healthy substitution for usual base fluids.

The fractional volume and thermal efficiency of the nano-sized floated particles set the characteristics of nanofluid heat transfer. Buongiorno provides a detailed overview of the thermal transmission of nanofluid and its main characteristics contributing to improved thermal transport performance^8^. Saleem et al.^9^ inspected the transport on naturally convected nanoliquid-filled substances. Through the remarkable study, the ethylene glycol-based hybrid nanoliquid was found to exert better thermal characteristics than that of nanofluid was provided by Nawaz et al.^10^. Mebarek-Oudina et al.^11^ investigated the response of entropy and convection in the case of a trapezoidal-shaped cavity filled with Cu-Al2O3/water hybrid nanofluid. The obtained findings indicated that increasing the concentration of nanoparticles enhances the heat transfer intensity inside the chamber. In the books^12–15^ and Journal papers^16–25^ it is possible to analyze more precisely the ongoing and future growth of nanofluids. Salimefendigil and Öztop^26^ analyzed the behavior of CuO–water nanofluid within 3D trapezoidal enclosure with a corrugated side under a uniform magnetic field. They found that increasing the number of corrugated waves leads to reduce the heat transfer efficiency. However, using CuO nanopowder had a favorable impact on energy transport. Fares et al.^27^ conducted a study of magneto-free convection inside a porous cavity filled with Al_2_O_3_-Cu/water hybrid nanofluid and provided with an adiabatic rotating cylinder. They noticed that the rise of Rayleigh number improves the heat transfer. However, the heat transfer is more controlled when Hartmann number is higher. Chamkha and Salimefendigil^28^ numerically examined the entropy and the free convection of Cu–water nanofluid carried inside a porous corrugated enclosure. They found that the rise of Grashof number enhances the heat transfer as well as increasing Darcy number. Moreover, they noticed that the frequency of corrugation waves had a significant impact when Grashof and Darcy numbers are important. Belhadj et al.^29^ investigated MHD natural convection of Ag–MgO/Water Hybrid Nanofluid inside a triangular cavity equipped with a rotating circular obstacle. The obtained results depicted that the growth in nanoparticles volume fraction improves the heat transfer. However, the presence of the magnetic field has a reducing impact on energy transmission. Fares et al.^30^ studied the response of the entropy generation of Ag–water nanofluid within a square-shaped enclosure in the presence of inclined magnetic forces. They found that the thermal performance of nanofluid increases with the augmentation of Rayleigh number and nanoparticles concentration. However, the inclination angle impacts the reducing effect of the magnetic field on the heat transport as well as the entropy generation. Salimefendigil and Öztop^31^ examined the entropy generation and the natural convection of an inclined cavity provided with an inner conductive curved partition and filled with nanofluid. They noticed that the total entropy generation rate decreases as Hartmann number increased. In addition, the contribution of solid and fluid domains varied as Hartmann number increased.

Theoretical, as well as practical considerations, make it necessary for mixed convection, which is normally caused in chambers loaded with higher energy elements with one or two sides. In reality, in different engineering applications, this configuration can be found. Many experiments have been reported on mixed convective flow in cavities to analyze thermal transport and hydraulic fluid in such geometries. In nature and a variety of handy transport process equipment, including furnaces, electrical cooling, solar collectors, and processing equipment, this phenomenon is significant. In this article, interest in commercial cooling cavities is determined by the problems found. The food items exposed to it are refreshed by new air circulation. Many experiments indicate poor cooling in the higher portion of the cavity. Thereby a strict temperature regulation of conservation in this region involves the existence of a non-durable commodity, Lei and Patterson^32^.

Reynolds et al.^33^ experimental and numerical investigated the case of a trapezoidal enclosure chamber replicates a solar-based thermal collector. The cavity's lower wall is translucent and has a directed solar-based flow. On the opposite, the upper wall simulates an exchanger, and experimental and computational findings indicate that there are recirculating cells that produce within the cavity isothermal zones. Sieres et al.^34^ carried out a computational investigation in a right angular triangular air-filled chamber class of laminated natural convectors along and in absence of surface radiation. For the maximal range of Rayleigh numbers (10^3^ and 10^6^), reduced slot angle, and increased slot radius, the average thermal transfer rate has been analyzed. Numerical analysis was performed at Hasnaoui et al.^35^ for dual valued Rayleigh numbers: 1.58 × 10^9^ and 5 × 10^10^, on the iso-celled chamber in a triangular shape loaded with the turbulent connected air in it. The findings of this survey indicate that the fluid is unstable and that the velocity and temperature vary sharply in a small stripe along the walls. This geometry, as compared to the square cavity, has high turbulence levels, but remains constant in the cavity center for a fixed Rayleigh number.

Mixed convective configuration on the flow field ensues while the forced flow dominates flow naturally induced flow with hotter fluids upstream movement along with the cooler fluids stays at the bottom. During the forced flow, shifting side walls or force injection phenomena, such as in lid drive flows or ventilated enclosures, cause the forced flow. Besides Prandtl ($Pr$) Grashof ($Gr$), Reynald number ($Re$) and their Richardson number ($Ri = Re/Gr^{2}$) are the dimensionless variables that represent the mixed convection. Flow situation became convectively forced for the values of Richardson number tends to zero. On other hand, it reflects the free convective flow behaviors spotted where Richardson number tends to infinity. Trending mixed convective flow studies over the closed chambers and cavity pull the researchers towards it with fascinating technical features. Louaraych et al.^36^.

The horizontal boundary motion and heat flow association in the rectangular chamber are investigating the combination of free and forced convection. Gangawane et al.^37^ studied for theoretical studies on the conduct of force flowing results in boundaries movement and natural convective flowing by built heated triangular blocks fitted with an enclosure. The numerical flow profile and characteristics of the thermal transport were studied by Salimipour^38^ due to the horizontal cylinders causing convective mixtures. The convective flow (free and force) in a ventilated parallelogram enclosure is studied by Gupta and Nayak^39^ and a stronger cold regime was observed due to the inflow of power through the contaminant source. A natural convective flowing result in a hot source on the ground, with forced convective stems shaping-wall motion of an enclosure, was analysed by Abu Hamdeh et al.^40^. In a second trial, Muhammad et al.^41^ tested the mixed convective flowing inside an enclosure with a warmer middle line in ethylene glycol-dependent nanofluid. Salimefendigil et al.^42^ conducted a numerical study to investigate MHD mixed convection inside a lid-driven chamber with a flexible fin and filled with CuO–water nanofluid. Their outputs showed that as the inclination angle of the magnetic field increases, the average Nusselt number declines. This impact is more evident in the cavity with the flexible fin.

The average number of Nusselt can be found to obey modification of the power law with the Rayleigh number. The study of Papanicolaou and Jaluria^43^ investigates the double waved rectangular chamber with lateral jet inputs. Unlike the previous example, its findings (represented for $Re = 100$) suggest that the flow-induced flow by a convective cell. Unprocessed air enters into the topper region of the chamber without influencing the rest.

Many different sectors of application have shown an interest in the mixed convection heat transfer and fluid movement that occurs in triangle enclosures. Solar engineering applications, electronic equipment cooling, geophysical fluid mechanics, and building and thermal insulation systems are just a few examples of what this technology may be used for. From the above literature review, the concluding remarks are: there has been substantial work done across the triangular aperture with lower lateral inlet and apply placed at the vertical outlet of the chamber which filled with efficient TiO_2_–SiO_2_/water hybrid nanofluid under the parametrical influence. The numerical analysis in the rectangular open cavity was analysed by Raji and Hasnaoui^44^. In HB configuration, their outcomes indicate that the thermal transport for the critical number of Reynolds ($Re_{c} = 100$) passes by limit corresponding to the absence of the recycling cells. Target of this works is to study and understanding the mixed convective flow behaviors under the impact of the numbers of Hartmann ($Ha$), Reynolds ($Re$) and Richardson ($Ri$). As a result, the current investigation is focused on the aforementioned goals.

## Mathematical formulation

**Figure 1:**
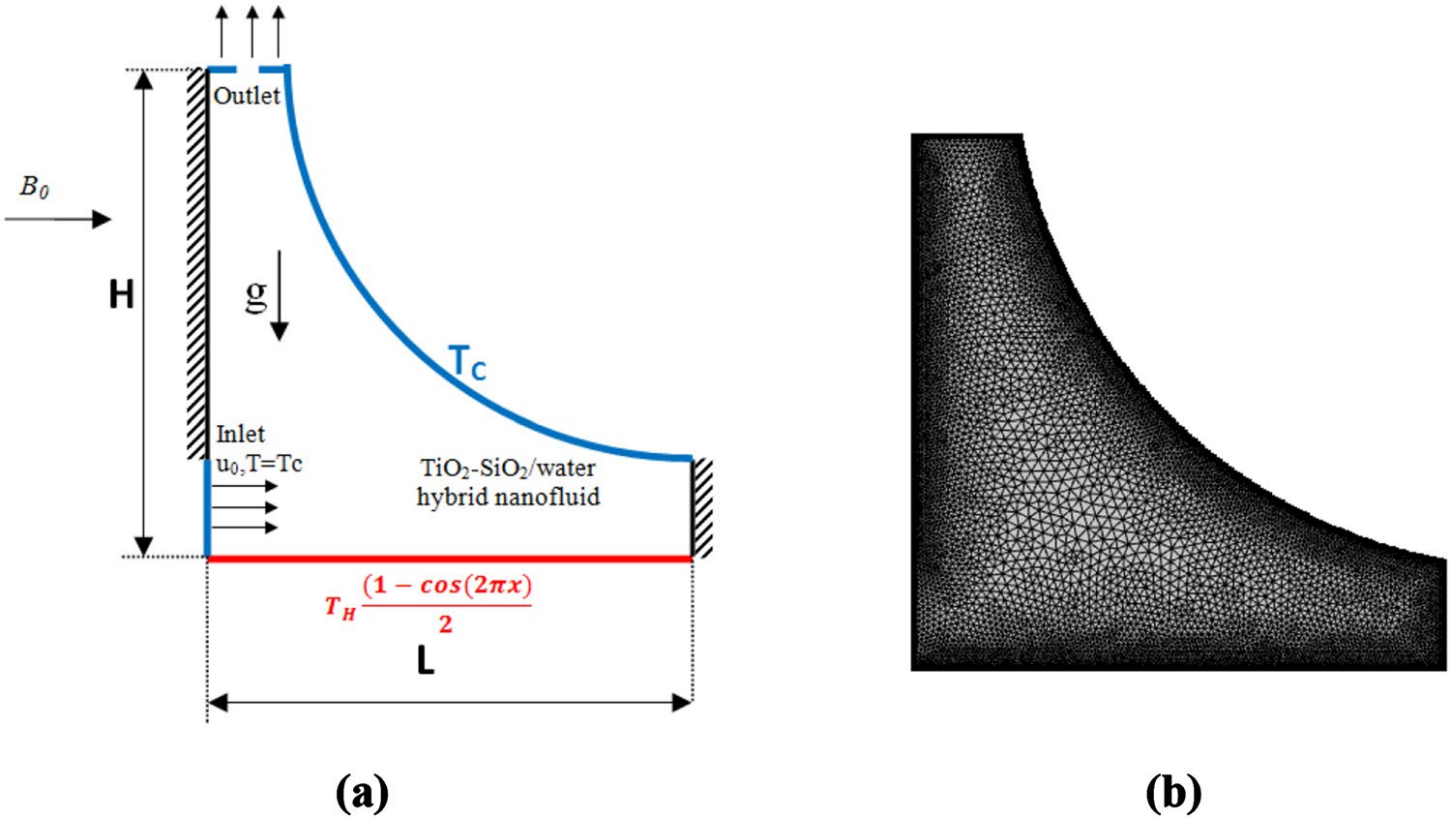
Vented cavity schematic representation with (**a**) boundary conditions (**b**) different configuration studies and mesh.

Figure 1 portrays the geometrical representation of the problem along with the generated mesh structures over the insulated triangular chamber apart from the heated bottom at a fixed temperature $T_{H}$ and inclined wall with cooling temperature $T_{c}$.

The triangular cavity is occupied with (TiO_2_–SiO_2_/water) nanofluid beneath the impact of a horizontal magnetic field. Except for the density variance which was formed by approximation of Boussinesq in the period of buoyancy, the thermo-physical aspects are supposed to be fixed. Thermo-physical features of hybrid-nanofluid at room temperature are offered in Table 1^45^. For a Newtonian fluid, laminar and stable-state flowing the continuity, motion in the Boussinesq approximation, and heat equations can be written in their nondimensional procedure as the following^46,47^:

1 $$\frac{\partial U}{{\partial X}} + \frac{\partial V}{{\partial Y}} = 0$$

2 $$U\frac{\partial U}{{\partial X}} + V\frac{\partial U}{{\partial Y}} = - \frac{\partial P}{{\partial X}} + \frac{{\rho_{bf} }}{{\rho_{hnf} }}\frac{{\mu_{hnf} }}{{\mu_{bf} }}\frac{1}{Re}\left( {\frac{{\partial^{2} U}}{{\partial X^{2} }} + \frac{{\partial^{2} U}}{{\partial Y^{2} }}} \right)$$

3 $$U\frac{\partial V}{{\partial X}} + V\frac{\partial V}{{\partial Y}} = - \frac{\partial P}{{\partial Y}} + \frac{{\rho_{bf} }}{{\rho_{hnf} }}\frac{{\mu_{hnf} }}{{\mu_{bf} }}\frac{1}{Re}\left( {\frac{{\partial^{2} V}}{{\partial X^{2} }} + \frac{{\partial^{2} V}}{{\partial Y^{2} }}} \right) + \frac{{\left( {\rho \beta } \right)_{hnf} }}{{\rho_{hnf} \beta_{bf} }}Ri\theta - \frac{{\rho_{f} }}{{\rho_{hnf} }}\frac{{Ha^{2} }}{Re}V$$

4 $$U\frac{\partial \theta }{{\partial X}} + V\frac{\partial \theta }{{\partial Y}} = \frac{{\sigma_{hnf} }}{{\sigma_{bf} }}\frac{1}{RePr}\left( {\frac{{\partial^{2} \theta }}{{\partial X^{2} }} + \frac{{\partial^{2} \theta }}{{\partial Y^{2} }}} \right)$$

**Table 1:** Thermo-physical aspects of the nanoparticles and the fluid (TiO_2_–SiO_2_/water) (50/50)^45,50–53^.

Physical properties	$c_{p} \left( {{\text{J/kg}}\;{\text{K}}} \right)$	$k\left( {{\text{W/m}}\;{\text{k}}} \right)$	$\rho \left( {{\text{kg/m}}^{3} } \right)$	$\beta \times { }10^{ - 5} \left( {{\text{K}}^{ - 1} } \right)$	$\sigma \left( {\text{S/cm}} \right)$
Water	4179	0.613	997.1	21	0.05
TiO_2_	686.2	8.954	4250	2.4	47
SiO_2_	745	1.4	2200	42.7	10^–12^

Designed for nanoparticles TiO_2_ and SiO_2_, the properties are acquired as^48^.

5 $$\varphi = \varphi_{{TiO_{2} }} + \varphi_{{SiO_{2} }}$$

6 $$\rho_{np} = \frac{{\varphi_{{TiO_{2} }} \rho_{{TiO_{2} }} + \varphi_{{SiO_{2} }} \rho_{{SiO_{2} }} }}{\varphi }$$

7 $$(c_{p} )_{np} = \frac{{\varphi_{{TiO_{2} }} (c_{p} )_{{TiO_{2} }} + \varphi_{{SiO_{2} }} (c_{p} )_{{SiO_{2} }} }}{\varphi }$$

8 $$\beta_{np} = \frac{{\varphi_{{TiO_{2} }} \beta_{{TiO_{2} }} + \varphi_{{SiO_{2} }} \beta_{{SiO_{2} }} }}{\varphi }$$

9 $$k_{np} = \frac{{\varphi_{{TiO_{2} }} k_{{TiO_{2} }} + \varphi_{{SiO_{2} }} k_{{SiO_{2} }} }}{\varphi }$$

10 $$\sigma_{np} = \frac{{\varphi_{{TiO_{2} }} \sigma_{{TiO_{2} }} + \varphi_{{SiO_{2} }} \sigma_{{SiO_{2} }} }}{\varphi }$$

The effectual dynamical viscidness depend on the Brinkmann mode is measured as^49^

11 $$\mu_{hnf} = \mu_{bf} \left( {1 + 32.795 \varphi - 7214 \varphi^{2} + 71400 \varphi^{3} - 0.1941 \times 10^{8} \varphi^{4} } \right)$$

12 $$\frac{{k_{hnf} }}{{k_{bf} }} = \frac{{0.1747 \times 10^{5} + \varphi }}{{0.1747 \times 10^{5} - 0.1498 \times 10^{6} \varphi + 0.1117 \times 10^{7} \varphi^{2} + 0.1997 \times 10^{8} \varphi^{3} }}$$

13 $$\frac{{\sigma_{hnf} }}{{\sigma_{bf} }} = 1 + \frac{{3\left[ {\left( {\frac{{\sigma_{np} }}{{\sigma_{bf} }}} \right) - 1} \right]\varphi }}{{\left( {\frac{{\sigma_{np} }}{{\sigma_{bf} }}} \right) + 2 - \left[ {\left( {\frac{{\sigma_{np} }}{{\sigma_{bf} }}} \right) - 1} \right]\varphi }}$$

Dimensionless numbers are written as:

14 $$\left. {\begin{array}{*{20}l} {X = \frac{x}{L},Y = \frac{y}{L},U = \frac{u.L}{{\alpha_{bf} }},V = \frac{v.L}{{\alpha_{bf} }}, \theta = \frac{{T - T_{C} }}{{T_{h} - T_{C} }},P = \frac{p}{{\rho_{hbf} U_{0}^{2} }}, Pr = \frac{{v_{bf} }}{{\alpha_{bf} }},Re = \frac{{\rho_{bf} U_{0} H}}{{\mu_{bf} }},} \hfill \\ {Ha = LB_{0} \sqrt {\frac{{\sigma_{hnf} }}{{\rho_{hbf} v_{bf} }}} ,Ri = \frac{{\beta_{bf} g\left( {T_{h} - T_{c} } \right)H^{3} }}{{U_{0}^{2} }}.} \hfill \\ \end{array} } \right\}$$

For the standard fluid and nanoparticles, Table 1 exhibits the thermo-physical aspects.

15 $$Nu_{loc} = - \frac{{k_{hnf} }}{{k_{bf} }}\frac{\partial \theta }{{\partial Y}}$$

16 $$Nu_{avg} = \frac{1}{L}\mathop \smallint \limits_{0}^{L} Nu_{loc} dL$$

The boundary conditions are:

- Inlet port: $$u = U_{0}$$, $$v = 0$$, $$T = T_{c}$$.
- Outlet port : $$\frac{\partial v}{{\partial y}} = \frac{\partial T}{{\partial y}} = 0$$.
- at the inclined curved cold wall: $u = v = 0$, $T = T_{c}$.
- at the adiabatic walls: $u = v = 0$, $\frac{\partial T}{{\partial y}} = \frac{\partial T}{{\partial x}} = 0$.
- at the bottom horizontal heater wall: $u = v = 0$, $T = T_{H} \frac{{(1 - cos\left( {2\pi x} \right)}}{2}.$

## Computational methodology

**Figure 2:**
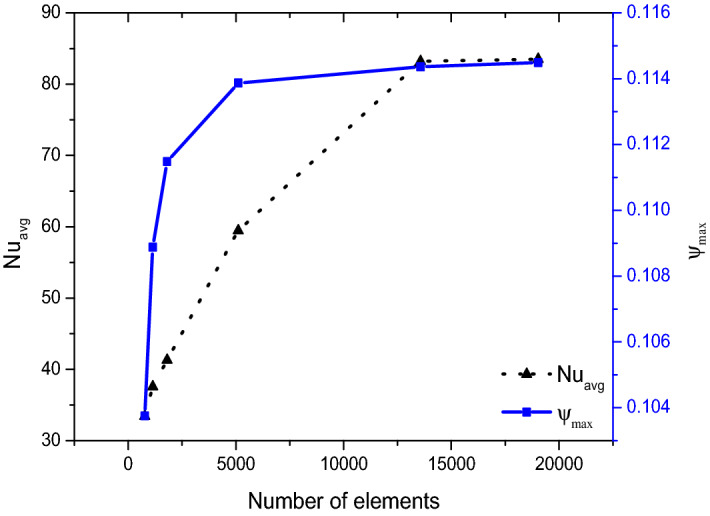
Validation with grid independence at $Re = 200$, $Ha = 0$, $Ri = 1$.

**Figure 3:**
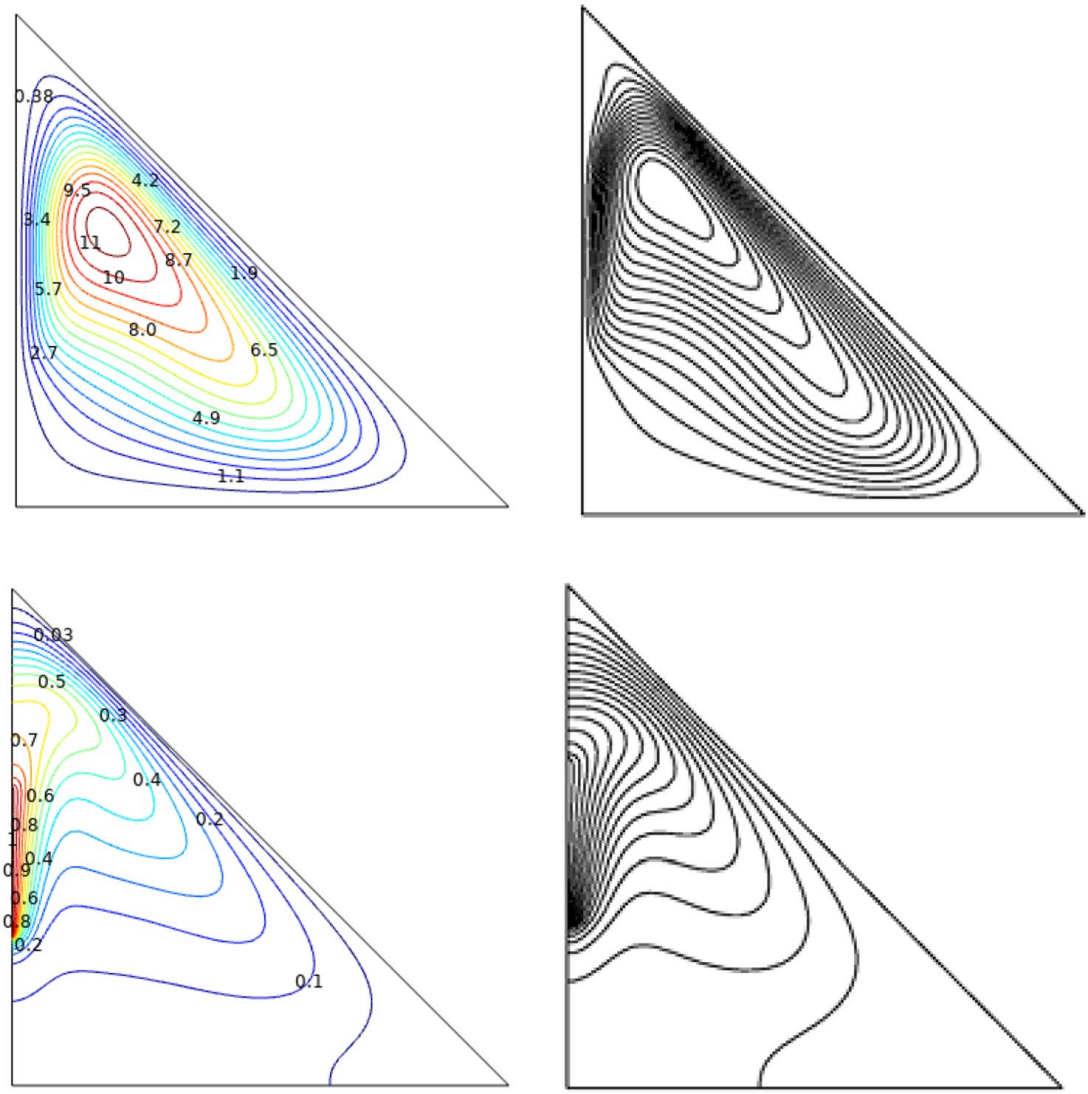
Comparing amongst the current study [right] and the outcomes of Ref.^54^ [left] at $Ra = 10^{6}$, $Ha = 0$.

Figure 2 evidences the result of grid autonomy analysis done for acquiring optimal count of grid elements to be adapted for mesh generation across the structure with 13,569 elements. Regarding validation of the numerical code engaged in this work were compared to results of^54^ and with that excellent agreement viewed in Fig. 3 this numerical model was employed for parametric analysis.

Modeled mathematical problems under existing situations were governed by the flow and thermal equations appendices with boundary situations. Efficient finite element technique combined with a weighted-residual process is engaged to track the solution. Geometry of the work has to be discretized with triangular elements used around 13,569 in the count. Error tolerance limit has been set to be below $\left| {\frac{{\Gamma^{ i + 1} - \Gamma ^{i} }}{{\Gamma ^{i} }}} \right| \le 10^{ - 6}$.

## Results discussion

Any substance enclosed by a solid wall, whether it's liquid or solid, is said to be an enclosure. Enclosures can be used to improve or slow down heat transmission, depending on the application. To ensure that electronic components operate at temperatures less than the critical temperature for the component, heat transfer in the enclosure must be increased. An enclosure that traps air between two panes, reduces the heat transmission from the inside. Solar collectors, electrical gadgets, and structures with slanted roofs all use triangular cavities. Flow and heat transmission study of TiO_2_–SiO_2_/water hybrid-nanofluid through the triangular chamber with single inlet in the bottom which is heated and outlet placed at the higher end has been carried out. Streamlines ($\Psi$) and isotherms ($T$) were plotted for various physical parameters like the numbers of Reynolds ($Re$), Richardson ($Ri$), and Hartmann ($Ha$). Through the grid-independent study, the optimal elements count of 13,569 elements was fixed. Numerical model was validated through the comparative study with Varol et al.^54^ which gives excellent agreement shown in Fig. 3.

### Effect of Reynolds number

**Figure 4:**
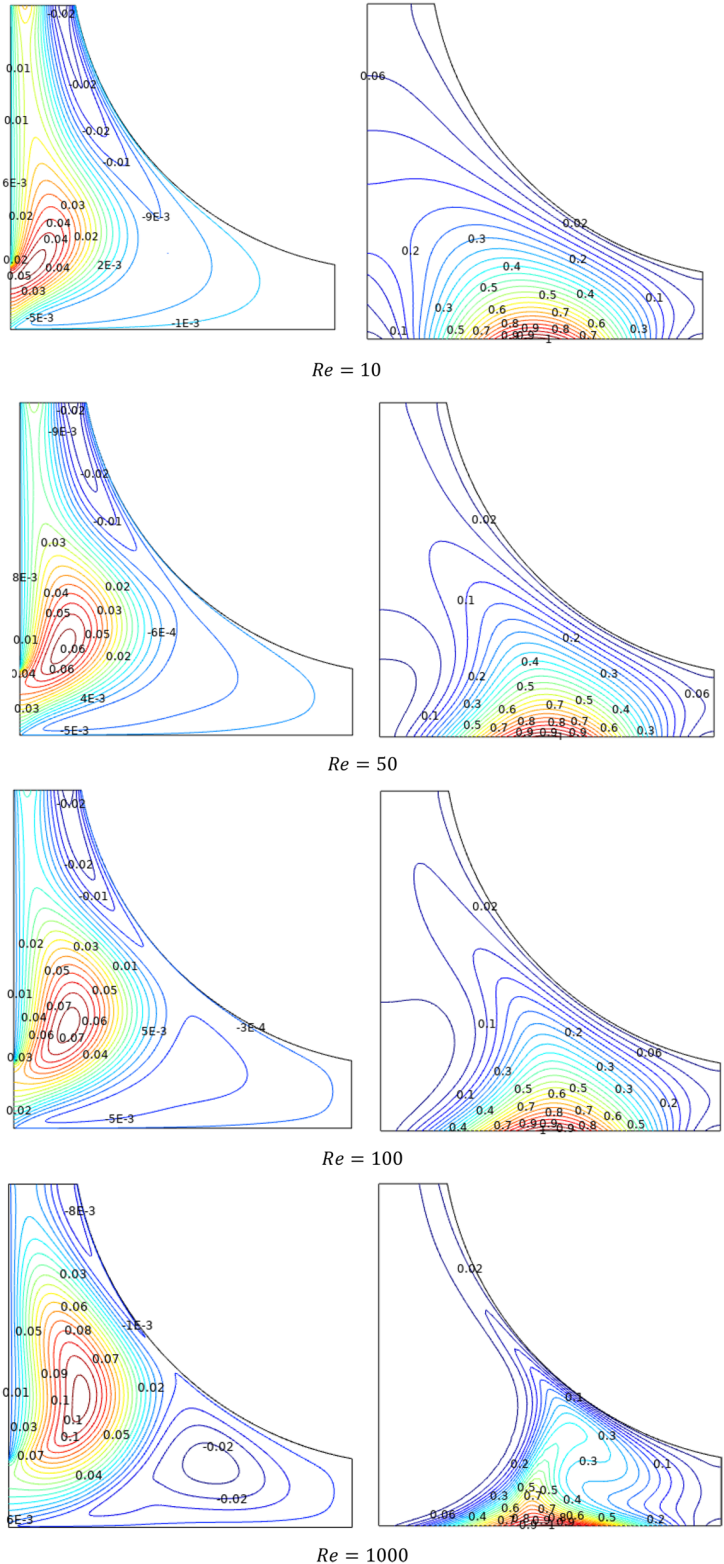
Variations of the streamlines ($\Psi$) and isotherms (*T*) with various Reyolds number ($Re$) at configuration 1, $Ha = 0$, $Ri = 1$, $\varphi = 0.02$.

Figure 4 conveys the behaviour of flow and thermal aspects in the schematized chamber for the Reynolds number ($Re$) through Streamlines ($\Psi$) and Isotherms ($T$) of TiO_2_–SiO_2_/water hybrid nanofluid respectively. For lower flow rates the streams started to convolute near the inlet and move deeper into the chamber assisted with the new contour in the father end for higher of $Re$ values. The isotherms replicate the thermal conditions across the chamber. As it is heated from below, the concentration of heat can be noted at the bottom and fades away as it propagates vertically towards an outlet. This due to the isotherms serves as exact replicas of the chamber's thermal constraints. Heat builds up at the bottom and dissipates as it moves upward to an outlet when heated from below. Regarding Reynolds number influences, as more the flow enters through the inlet for hiked values of $Re$, the isotherms were blown towards the edge.

**Figure 5:**
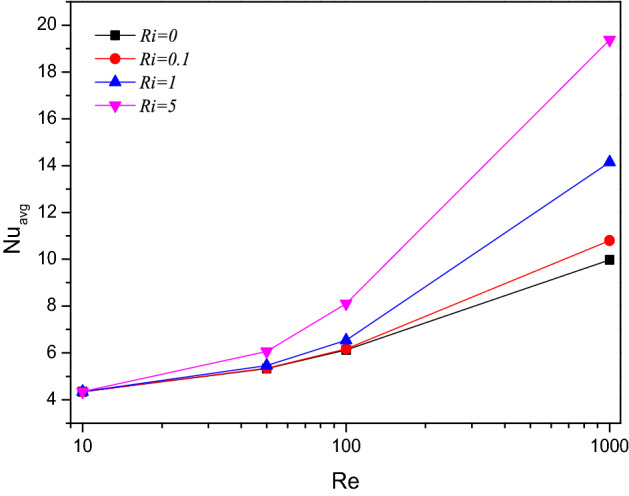
Variations of $Nu_{avg}$ with various $Re$ for $Ri$ variations, $Re = 1000$, $\varphi = 0.02$.

Nusselt number plotting for $Re$ and $Ri$ were characterized by the thermal transporting rate by the Nusselt number ($Nu$) through Fig. 5. In both cases, the heat transmission efficiency of TiO_2_–SiO_2_/water hybrid-nanofluid gets improved flow rate as well convection rate respectively for higher amounts of $Re$ and $Ri$.

**Figure 6:**
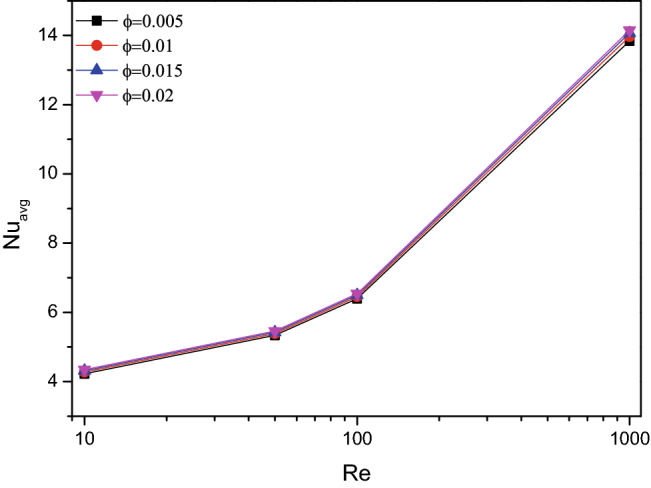
Variations of $Nu_{avg}$ with various $Re$ for solid volume fraction variations ($\varphi$), $Re = 1000$, $Ri = 1$.

**Figure 7:**
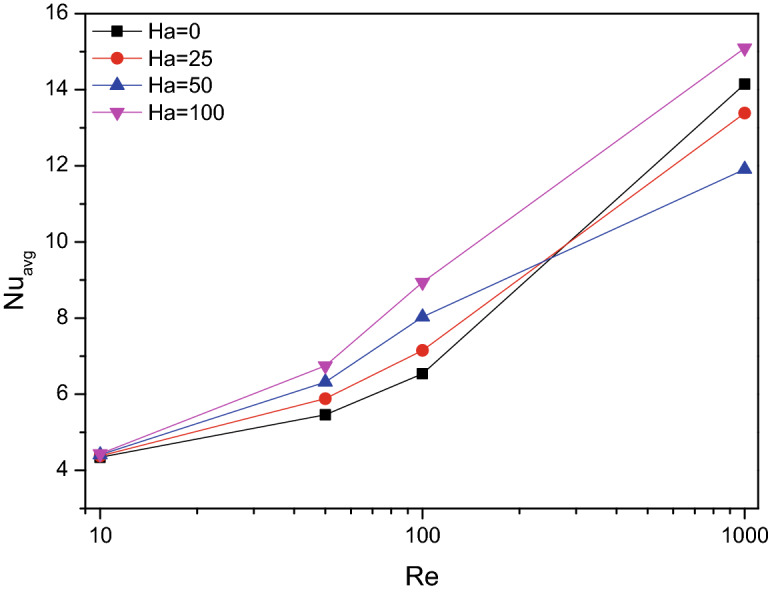
Variations of $Nu_{avg}$ with various $Re$ for $Ha$ variations, $Re = 1000$, $\varphi = 0.02$.

Figures 6 and 7 disclose the impact of fractional volume ($\varphi$) and Hartmann number ($Ha$) combined with Reynolds number ($Re$) over the thermal transferring rate. As the particle fraction increased the thermal transferring efficiency of TiO_2_–SiO_2_/water hybrid nanofluid, the Hartmann number ($Ha$) exerts fluctuation in thermal transfer rate for higher values of Reynolds number ($Re$). This may be due to the dual effect of magnetic interaction and the flow turbulence accord in the chamber flow. This effect of “fluctuating” is due to the interaction between the flow velocity and the applied magnetic field.

### Effect of Richardson number

Indent of this works is to analyze the mixed convective facets of TiO_2_–SiO_2_/water hybrid-nanofluid. Richardson number ($Ri$) plays a noteworthy part in manipulating the convection features of the flow stream. Curtailed values of Richardson number ($Ri$) reflect the forced convection ($Ri < 0.1$) followed by higher values for the mixed convective flowing ($0.1 < Ri < 10$) and free convective flowing ($Ri > 10$).

**Figure 8:**
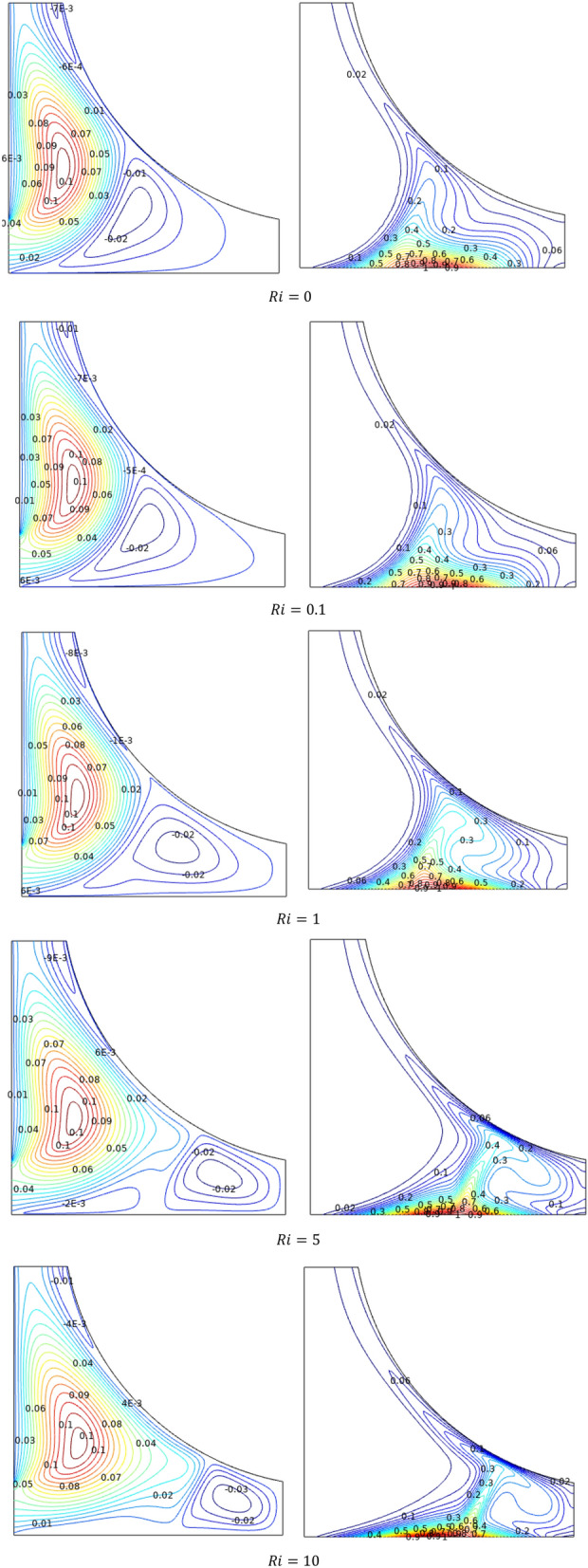
Variations of the streamlines ($\Psi$) and isotherms (*T*) with various $Ri$ at configuration 1, $Re = 1000$, $Ha = 0$, $\varphi = 0.02$.

Streamlines ($\Psi$) and isotherms ($T$) for Richardson number ($Ri$) variations were portrayed in Fig. 8. Under the forced and mixed convection stages, the streams in the chamber attain two contours, one of which dominates over the other to spreads wider and shrinks the outer contour, it might be due to crucial buoyancy alterations through ($Ri$) in TiO_2_–SiO_2_/water hybrid nanofluid. Such coiled streams tend to swipe away the temperature from the chamber which is evident through the spiraled isotherms in the farther end of the chamber. Initially, for lower values of $Ri$, the isotherms were elevated vertically towards the outlet.

**Figure 9:**
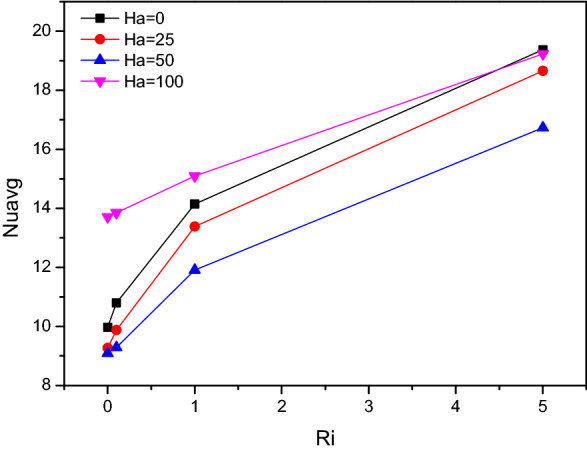
Variations of $Nu_{avg}$ with various $Ri$ for $Ha$ variations, $Re = 1000$, $\varphi = 0.02$.

**Figure 10:**
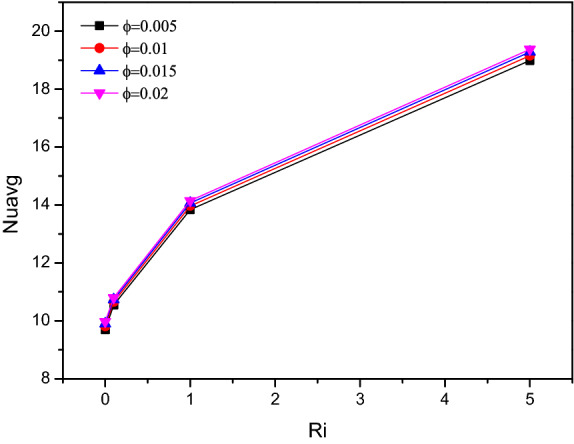
Variations of $Nu_{avg}$ with various $Ri$ for solid volume fraction variations ($\varphi$), $Re = 1000$, $Ri = 1$.

Average Nusselt number ($Nu_{avg}$) for altering Richardson number in combination with Hartmann number ($Ha$) and fractional volume ($\varphi$) were crafted in Figs. 9 and 10 respectively. The thermal transferring rate gets hiked for higher values of $Ri$, especially after $Ri = 1$ which represents the vital impact of mixed convection flow behaviour. Simultaneously for both increasing values of fractional volume ($\varphi$) and Hartmann number ($Ha$), the average Nusselt number ($Nu_{avg}$) gets increased. Notably for larger amounts of $Ha$ corresponds to mixed convective mode, attains few fluctuations in the average Nusselt number ($Nu_{avg}$).

### Effect of Hartmann number

**Figure 11:**
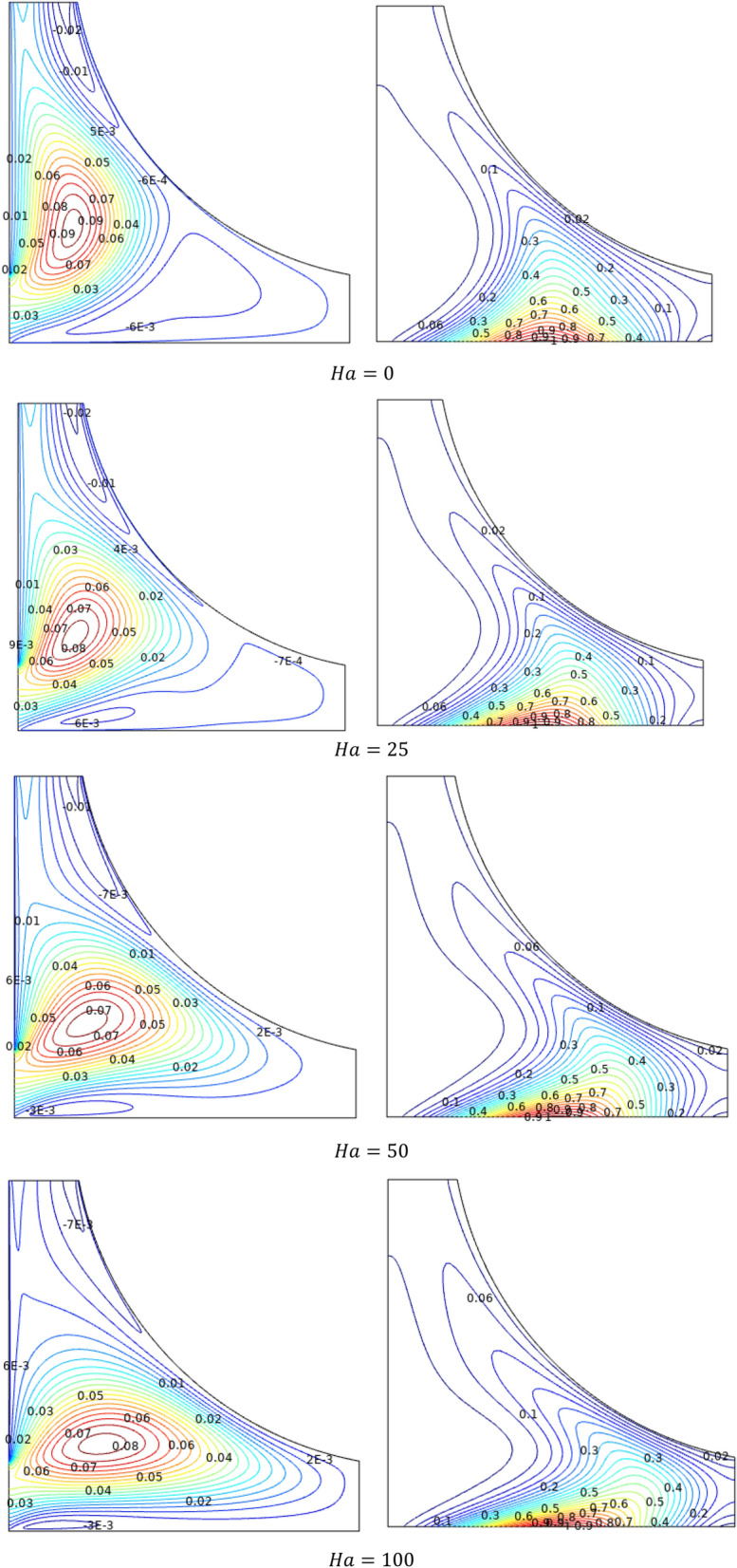
Variations of the streamlines ($\Psi$) and isotherms (*T*) with various $Ha$ at configuration 1, $Re = 200$, $Ri = 1$, $\varphi = 0.02$.

Figure 11 elucidates the magnetic interaction effects over the streamlines ($\Psi$) and isotherms ($T$) through the Hartmann number ($Ha$). Lofted values of Hartmann number ($Ha$), breaks in stream flow velocity with the opposing magnetic fields. Initially, forced streams to enter into the chamber to form two clear contours, as the Hartmann number increases, the flow slows down and the inlet contour covers the chamber due to smooth entry of fluid. Similar trend can be found in isotherms also. Slower flow absorbs more heat from the chamber wall, as evidenced by the structural shift in isotherms from spiral to stretched shape along the bottom wall.

**Figure 12:**
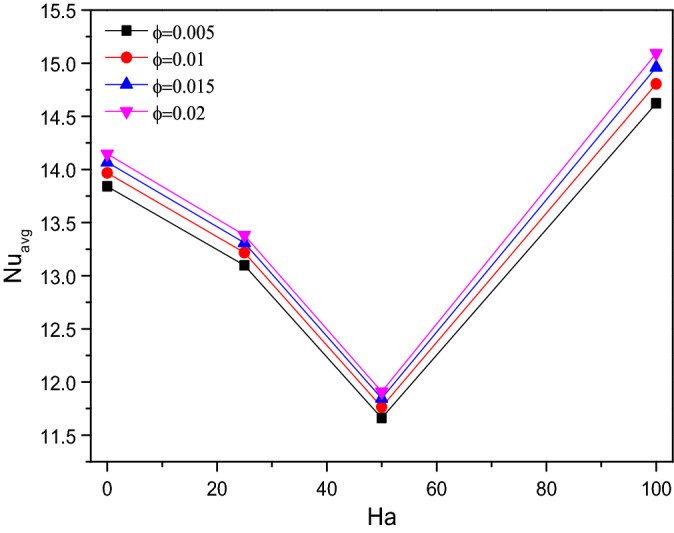
Variations of $Nu_{avg}$ with various $Ha$ for $\varphi$ variations, $Re = 1000$, $Ri = 1$.

Average Nusselt number ($Nu_{avg}$) of TiO_2_–SiO_2_/water hybrid nanofluid were plotted against the Hartmann number ($Ha$) for increasing values of fractional volume ($\varphi$) in Fig. 12. As the strength of hybrid nanofluid increased by additional volume fraction of solid particles, the average Nusselt number ($Nu_{avg}$) gets improved. An increase in thermal conductivity boosts heat transfer efficiency. Nanoparticles and random fluid motion within the base fluid may both help to reduce the thermal boundary layer thickness, which may in turn aid in heat transfer improvement. In terms of Hartmann number ($Ha$), it gets decelerates up to some extent near $Ha = 50$. Later it gets enhanced for higher values of Hartmann number ($Ha$).

## Conclusion

Planned work on the triangular chamber with a single inlet in the bottom which is heated and outlet placed at the higher end underwent by the flow of TiO_2_–SiO_2_/water hybrid nanofluid has been carried out. Flow nature and thermal actions were modeled by the Streamlines ($\Psi$) and isotherms ($T$) respectively. Outcomes were plotted against crucial physical parameters like the numbers of Reynolds ($Re$), Richardson ($Ri$), and Hartmann ($Ha$). Validation of the working procedure has been done through the comparative study of a numerical model with Varol et al.^51^. Optimal mesh count was also obtained with the grid-independent study of nearly 13,569 elements.

Streamlines ($\Psi$) contours reflect the inflow to the triangular chamber, as part of the flow hits the further end and forms a smaller counter and remaining flows wipes upward towards the outlet was characterized by stretches in the contours towards the outlet region for all the physical parameters like $Re$, $Ri$, and $Ha$.

Isotherms ($T$) portrays the thermal movements inside the triangular chamber flowed with the TiO_2_–SiO_2_/water hybrid nanofluid. Since the chamber is heated from the bottom, the isothermal plots exhibit the bottom-based thermal contours. Due to inflow fluid, the contours bend outward to the inlet of the chamber and elevates towards the outlet trays around the opposite wall of the chamber to the inlet.

Plots of average Nusselt number ($Nu_{avg}$) depicts the heat transferring process held across the chamber. It gets increased for higher values of $Re$, $Ri$, $\varphi$ and $Ha$. Especially, for Hartmann number ($Ha$) during higher values it gets fluctuates for values ($Ha < 100$).

## Data Availability

The results of this study are available only within the paper to support the data.

## List of symbols

$c_{p}$: Specific heat capacity (${\text{J}}\;{\text{kg}}^{ - 1} \;{\text{K}}^{ - 1}$)
$${\text{g}}$$: Gravitational acceleration ($${\text{m}}\;{\text{s}}^{ - 2}$$)
$H,L$: Dimensionlal of triangilar cavity (m)
$$k$$: Thermal conductivity ($${\text{W}}\;{\text{m}}^{ - 1} \;{\text{K}}^{ - 1}$$)
$$p$$: Pressure ($${\text{Pa}}$$)
$$P$$: Dimensionless pressure
$$T$$: Temperature (K)
$$u, v$$: Velocity components ($${\text{m}}\;{\text{s}}^{ - 1}$$)
$U, V$: Dimensionless velocity components
$x, y$: Dimensional Cartesian coordinates (m)
$X, Y$: Dimensionless Cartesian coordinates
$$Ha$$: Hartmann number
$$Nu$$: Nusselt number
$$Pr$$: Prandtl number
$$Ri$$: Richardson number
$$Re$$: Reynolds number
$$u,v$$: Velocity components
$U,V$: Nondimensional velocity components
$$x,y$$: Coordinate
$B_{0}$: Intensity of magnetic field

## Greeks symbols

$$\alpha$$: Thermal diffusivity
$$\nu$$: Momentum diffusivity
$$\varphi$$: Volume fraction
$\beta$: Thermal expansion coefficient
$$\mu$$: Dynamic viscosity
$$\rho$$: Density
$$\sigma$$: Electrical conductivity
$$\theta$$: Dimensionless temperature
$\psi$: Non-dimensional stream function

## Subscripts

$$f$$: Fluid
$$hnf$$: Hybrid nanofluid
$$np$$: Solid particles
$$avg$$: Average
$$loc$$: Local
$$h$$: Hot
$$c$$: Cold
